# Different prophylactic measures for preventing postoperative deep venous thromboembolism in adenomyosis: a retrospective study

**DOI:** 10.1016/j.clinsp.2025.100700

**Published:** 2025-06-14

**Authors:** Yan Lei, Na Chen, Yuqin Tang, Xiaojia Xie

**Affiliations:** Hubei Province Women and Children Hospital, Wuhan, PR China

**Keywords:** Adenomyosis, Coagulation function, Preventive measures for thrombosis, Venous thromboembolism, Laparoscopy, Adenomyosis focus excision

## Abstract

**Objective:**

To investigate the incidence of postoperative venous thromboembolism in *women* with uterine adenomyosis treated using different surgical methods, and to evaluate the associated clinical and laboratory parameters, including thromboembolism assessment after laparoscopic excision of adenomyosis.

**Methods:**

(1) A retrospective collection of clinical data was conducted for 411 women with uterine adenomyosis and 33 women without adenomyosis who underwent surgical treatment at Hubei Provincial Maternal and Child Healthcare Hospital from March 2020 to December 2022. The clinical data, surgical methods, and preoperative and postoperative coagulation parameters were compared between the two groups. (2) The incidence of venous thromboembolism in women with and without uterine adenomyosis was analyzed. (3) The differences in the incidence of venous thromboembolism among women with uterine adenomyosis who underwent different surgical methods were analyzed, including clinical data and preoperative and postoperative coagulation parameters of women who did not experience venous thromboembolism. (4) The incidence of venous thromboembolism was compared between women receiving mechanical thromboprophylaxis and those receiving a combination of mechanical and pharmacological prophylaxis.

**Results:**

(1) Comparative analysis of clinical data showed that women with uterine adenomyosis had fewer pregnancies and births, lower hemoglobin levels, and larger uterine volumes compared to women without adenomyosis (*p* < 0.05). There were no significant differences in preoperative and postoperative coagulation parameters between the two groups (*p* > 0.05). (2) The preoperative platelet count and postoperative d-dimer levels were higher in women with uterine adenomyosis than in those without adenomyosis (*p* < 0.05). (3) The incidence of venous thromboembolism was higher in the uterine adenomyosis group (9.78 %) than in the non-adenomyosis group (3.03 %). Among the women with uterine adenomyosis, the incidence of venous thromboembolism after laparoscopic lesion excision (18.46 %) was higher than that after laparoscopic hysterectomy (7.86 %). (4) Compared with the mechanical prophylaxis group, the mechanical plus pharmacological prophylaxis group had a significantly lower incidence of venous thromboembolism [18.52 % (15/81) vs. 7.62 % (25/328)], with a statistically significant difference (*p* < 0.05).

**Conclusion:**

The incidence of postoperative venous thromboembolism is relatively high in women with uterine adenomyosis, particularly in those who underwent laparoscopic lesion excision. Even in the absence of typical symptoms and despite thromboprophylaxis, screening for venous thromboembolism should be considered within 7 days postoperatively.

## Highlights

• High postoperative VTE incidence in women with adenomyosis after surgery.
• Laparoscopic lesion excision shows higher VTE rates than hysterectomy.
• Combined mechanical and pharmacological prophylaxis reduces VTE incidence.
• Preoperative d-dimer levels significantly elevated in VTE cases.
• The study emphasizes the need for VTE screening post-adenomyosis surgery.

## Abbreviations

VTE: Venous Thromboembolism
DVT: Deep Vein Thrombosis
PE: Pulmonary Embolism
AM: Adenomyosis
CIN: Cervical Intraepithelial Neoplasia
Hb: Hemoglobin
PLT: Platelet Count
PT: Prothrombin Time
APTT: Activated Partial Thromboplastin Time
FIB: Fibrinogen
TT: Thrombin Time
CTPA: Computed Tomographic Pulmonary Angiography
BMI: Body Mass Index.

## Introduction

The incidence of Venous Thromboembolism (VTE) after gynecological surgery has garnered increasing attention. VTE includes Deep Vein Thrombosis (DVT) and Pulmonary Embolism (PE). Studies have indicated that the incidence of DVT during the perioperative period in women with benign gynecological conditions ranges from 10 % to 15 %, whereas it can be as high as 19.6 % to 38 % in women with malignant gynecological tumors.^1^ Women with DVT may present with clinical symptoms such as lower limb pain, swelling, edema, erythema, fever, superficial venous dilation, or tenderness; however, some women may exhibit atypical clinical symptoms, making it challenging to diagnose VTE based solely on clinical presentation, necessitating imaging studies for a definitive diagnosis.^2^ DVT is the primary source of thrombi in PE, which often coexists with DVT. Therefore, if a woman experiences sudden unexplained shortness of breath, hemoptysis, chest pain, unexplained heart failure, or shock after gynecological surgery, there should be a high index of suspicion for the possibility of PE. Notably, some women diagnosed with PE may already have asymptomatic DVT in their lower limbs,^3^ and the occurrence of PE can be rapid and fatal. Due to factors such as prolonged surgical duration, significant trauma, extended postoperative bed rest, and older age, surgery for malignant gynecological tumors has been the primary focus for the prevention and screening of VTE after gynecological surgery, whereas postoperative VTE screening for other benign gynecological conditions has received comparatively less attention.

Adenomyosis (AM) is a benign gynecological condition characterized by the invasion of the endometrium (including glands and stroma) into the myometrium. The main clinical symptoms include menorrhagia (which can lead to severe anemia), dysmenorrhea, and infertility, significantly affecting physical and mental health.^4^ AM is prevalent among women of reproductive age, with an incidence rate of 20.9 % to 34 %.^5^, ^6^, ^7^ Non-steroidal anti-inflammatory drugs, progestins, and combined oral contraceptives can alleviate pain and reduce bleeding and are used for the conservative treatment of AM; however, the effects of medication are temporary, and symptoms may recur after discontinuation.^8^, ^9^, ^10^, ^11^

When women with AM experience dysmenorrhea, excessive menstrual bleeding, treatment failure with medications, or contraindications to drug therapy, total hysterectomy or AM lesion excision may be considered as treatment options. Literature has reported cases of VTE and cerebral infarction in women with AM who did not undergo surgery, suggesting that the coagulation function in these women may be abnormal.^12^, ^13^, ^14^, ^15^, ^16^, ^17^, ^18^, ^19^, ^20^, ^21^, ^22^ Surgery may increase the risk of thrombosis because reduced preoperative mobility, intraoperative immobilization, and prolonged postoperative bed rest can significantly slow venous blood flow. Anesthesia and surgical trauma promote the release of tissue factors and directly activate the extrinsic coagulation pathway, leading to a hypercoagulable state or thrombosis. Without preventive measures, the incidence of DVT in women undergoing surgical procedures ranges from 10 % to 40 %.^23^ Currently, there are a few large-sample reports on the incidence of VTE after surgical treatment in women with AM. The present study analyzed the incidence of VTE in women with AM treated using different surgical methods and compared the incidence of VTE in women with and without AM undergoing total hysterectomy. The authors also analyzed various clinical characteristics of the study participants and compared the incidence rates of VTE among the different groups. The authors hope that these findings will raise awareness among clinicians regarding early diagnosis, prevention, and treatment of thrombosis after AM surgery.

## Materials and methods

### Patients and clinical data

Clinical data were retrospectively collected from 411 women with AM and 31 women who underwent total hysterectomy for Cervical Intraepithelial Neoplasia III (CIN III) at Hubei Provincial Maternal and Child Healthcare Hospital from March 2020 to December 2022. CIN III, a precancerous lesion of the cervix, typically does not involve lesions in the myometrium, and there are usually no changes in uterine volume or menstrual flow. For women with CIN III without fertility requirements, total hysterectomy can be performed, and it is more suitable than other benign gynecological conditions for comparative studies on total hysterectomy in women with AM. Venous blood samples were collected from all women to assess complete blood counts and coagulation parameters, along with clinical data. Complete blood count parameters included Hemoglobin (Hb) and Platelet Count (PLT), whereas coagulation parameters included Prothrombin Time (PT), Activated Partial Thromboplastin Time (APTT), Fibrinogen (FIB), Thrombin Time (TT), and d-dimer levels. Uterine volume was calculated based on transvaginal (or transrectal) ultrasound reports obtained within one month prior to inclusion in the study using the formula V=length×width×thickness×0.532. This study was approved by the Medical Ethics Committee of Hubei Provincial Maternal and Child Healthcare Hospital (n° IEC042).

### Inclusion and exclusion criteria

The inclusion criteria were as follows: (1) Open or laparoscopic surgery and (2) No use of hemostatic, anticoagulant, or steroid hormones within three months prior to surgery. Women diagnosed with malignant tumors postoperatively, those with hematological disorders or coagulation dysfunction, those experiencing acute or chronic pelvic inflammatory disease, those who refused VTE screening, and those who declined to participate in this study were excluded.

### Groups

Women were grouped by disease into the AM group (*n* = 411) and the non-AM group (*n* = 31), which consisted of women with CIN III confirmed by preoperative ultrasound and postoperative pathological examination showing no lesions in the myometrium. They were further divided by surgical method into laparoscopic total hysterectomy (*n* = 318), abdominal total hysterectomy (*n* = 9), laparoscopic lesion excision (*n* = 65), and abdominal lesion excision (*n* = 17) groups. Two women were screened for VTE due to significantly elevated preoperative d-dimer levels and did not undergo surgery.

Women were also grouped by the thromboprophylaxis method. According to the Caprini risk assessment model,^24^ all surgical women had a thrombotic risk score of 3‒5, categorizing them as high to very high risk. All the women required postoperative VTE prophylaxis. Preventive measures were stratified based on the risk of postoperative active bleeding: the mechanical prophylaxis group included women undergoing lesion excision who received intermittent pneumatic compression during surgery and graduated compression stockings postoperatively to prevent VTE, without the use of anticoagulants. The mechanical plus pharmacological prophylaxis group consisted of women undergoing total hysterectomy who had a low risk of rebleeding; therefore, they received subcutaneous low-molecular-weight heparin starting at 12 h postoperatively for thromboprophylaxis.^25^

### Diagnostic criteria

The diagnostic criteria for AM included clinical symptoms, such as dysmenorrhea and increased menstrual flow, with imaging studies suggesting AM and postoperative histopathological confirmation of AM. The diagnostic criteria for CIN III involve a histological examination showing severe atypical hyperplasia of the cervix, significant cellular atypia, loss of polarity, and abnormal proliferation of cervical cells extending into more than two-thirds of the epithelial layer.^26^ None of the women with CIN III included in the study had postoperative pathology upgraded to invasive cervical cancer.

The diagnostic criteria for VTE required all women, regardless of symptoms, to undergo bilateral lower limb venous Doppler ultrasound within 2‒7 days postoperatively to screen for DVT. The diagnostic criteria for DVT included venous lumen dilation; loss of compressibility; absence of blood flow signals or filling defects; and a lack of enhancement, weakening, or disappearance of blood flow signals upon distal limb compression. Women diagnosed with DVT, regardless of the presence of typical PE symptoms, underwent further Computed Tomographic Pulmonary Angiography (CTPA) to screen for PE. The diagnostic criteria for PE were based on CTPA findings showing filling defects within the pulmonary arteries, either partially or completely surrounded by opacified blood flow (rail sign) or complete filling defects with non-visualization of distal vessels.^27^

### Statistical methods

Statistical analyses were performed using SPSS version 22.0. Continuous variables that followed a normal distribution were expressed as x̄ ± s, and comparisons were made using a one-way analysis of variance. For variables that did not follow a normal distribution, data were expressed as median (25 %‒75 %) [M (P25‒P75)], and comparisons were made using non-parametric tests. Categorical variables were presented as counts and percentages, with comparisons made using the Chi-Square (χ²) test; *p* < 0.05 was considered statistically significant.

## Results

### Comparison of clinical data between women with AM and non-AM

General characteristics were compared between the 411 women with AM and 33 women without AM. The results indicated that age, number of pregnancies, number of live births, and hemoglobin levels were significantly lower in women with AM than in those without non-AM women (*p* < 0.05). Additionally, the uterine volume in women with AM was significantly greater than that in women, with a statistically significant difference (*p* < 0.001). No statistically significant differences were observed between the two groups in terms of the number of miscarriages or Body Mass Index (BMI) (*p* > 0.05) (Table 1).

**Table 1:** Comparison of general data between patients with AM and non-AM.

Clinical features	AM (*n* = 411)	non-AM (*n* = 33)	Statistic	p
Age (years, x̄ ± *s*)	46.4 ± 5.3	56.7 ± 7.1	*F* = 111.364	<0.001
Pregnancy ([M (P25‒P75)])	3.0 (2.0‒4.0)	4.0 (3.0‒4.0)	*Z* = −2.606	0.009
Yield [M (P25‒P75)]	1.0 (1.0‒2.0)	2.0 (1.0‒3.0)	*Z* = −4.003	<0.001
Abortion ([M (P25‒P75)])	2.0 (1.0‒3.0)	2.0 (1.0‒3.0)	*Z* = −0.138	0.890
BMI (kg/m^2^, x̄ ± *s*)	23.8 ± 2.8	23.4 ± 3.2	*F* = 0.516	0.473
Uterine Volume [cm^3^, (P25‒P75)]	428.59 (276.08‒640.0)	36.00 (36.00‒60.00)	*Z* = −9.364	<0.001
Hb [g/L, M (P 25‒P75)]	106.00 (87.50‒123.00)	124.00 (116.00‒131.00)	*Z* = −4.139	<0.001

AM, Adenomyosis; Hb, Hemoglobin.

### Comparison of coagulation parameters before and after surgery between women with AM and non-AM

The authors compared the coagulation parameters in women who underwent the same surgical procedure (laparoscopic total hysterectomy). The results showed that women with AM had significantly higher PLT and elevated postoperative d-dimer levels than women without AM (*p* < 0.05). However, there were no statistically significant differences in TT, PT, APTT, FIB, or preoperative d-dimer levels between the two groups (*p* > 0.05) (Table 2).

**Table 2:** Comparison of preoperative and postoperative coagulation indexes between patients with AM and non-AM [M (P25‒P75)].

Indicators	AM (*n* = 318)	Non-AM (*n* = 31)	Z	p
PLT (10^9/L^)	257.00 (215.00‒305.50)	215.00 (181.50‒272.50)	−3.052	0.002
TT (S)	15.20 (14.50‒16.05)	15.10 (14.70‒15.75)	−0.252	0.801
PT (S)	11.50 (11.00‒12.10)	11.40 (11.00‒11.95)	−0.568	0.570
APTT (S)	29.60 (27.60‒31.70)	29.60 (27.20‒31.40)	−0.193	0.847
FIB (g/L)	2.55 (2.27‒2.87)	2.70 (2.39‒2.88)	−1.291	0.197
Preoperative d-dimer (µg/mL)	0.27 (0.20‒0.42)	0.28 (0.16‒0.40)	−0.746	0.456
Postoperative d-dimer (µg/mL)	1.27 (0.94‒1.91)	1.01 (0.71‒1.65)	−2.473	0.013

AM, Adenomyosis; PLT, Platelet Count; PT, Prothrombin time; APTT, Activated Partial Thromboplastin Time; FIB, Fibrinogen; TT, Thrombin Time.

### Incidence of postoperative VTE in women with AM compared to non-AM

**Fig. 1:**
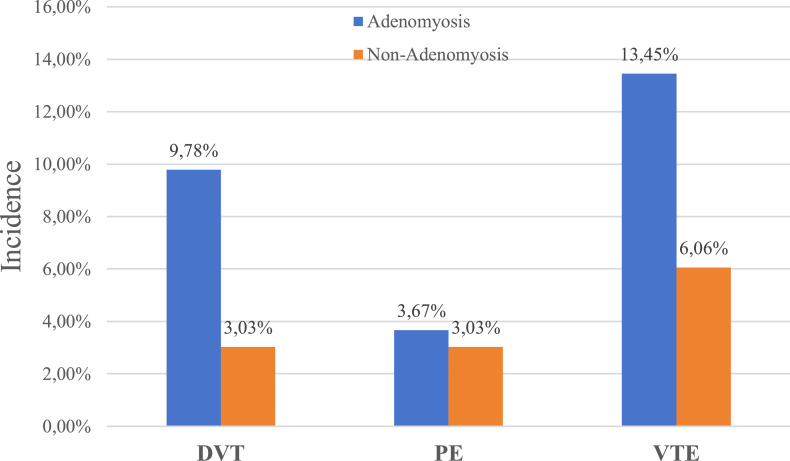
**Incidence of deep vein thrombosis by disease category.** VTE, Venous Thromboembolism; DVT, Deep Vein Thrombosis; PE, Pulmonary Embolism.

Comparing based on disease category, the incidence of DVT was higher in the AM group at 9.78 % (40/409) compared to the non-AM group at 3.03 % (1/33). The incidence of PE was also higher in the AM group (3.67 % [15/409] vs. 3.03 % [1/33], respectively). Overall, the incidence of VTE was higher in the AM group at 13.45 % (55/409) compared to 6.06 % (2/33) in the non-AM group (Fig. 1).

**Fig. 2:**
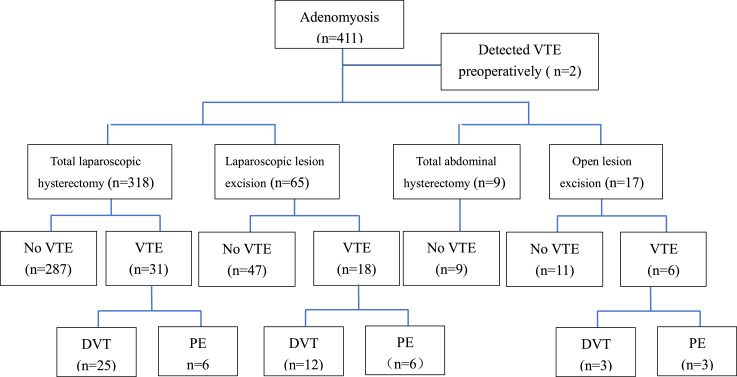
**TVE screening process for Adenomyosis patients.** VTE, Venous Thromboembolism; DVT, Deep Venous Thrombosis; PE, Pulmonary Embolism.

**Fig. 3:**
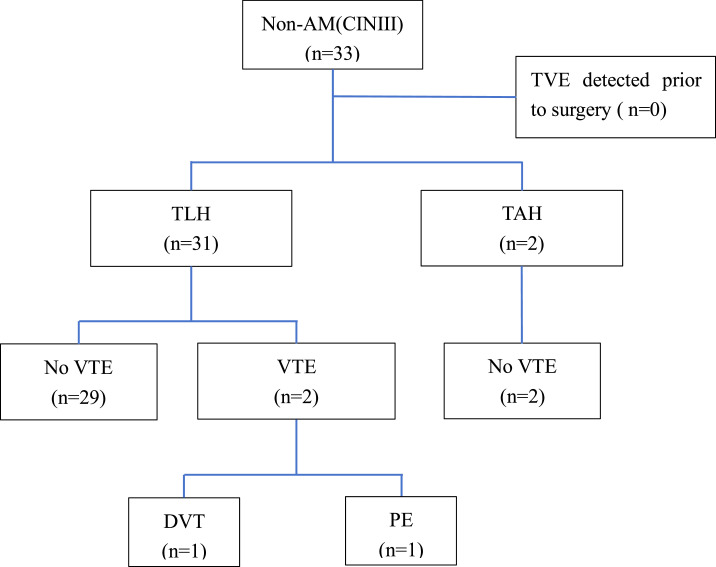
**TVE screening process for non-AM patients.** VTE, Venous thromboembolism; DVT, Deep Venous Thrombosis; PE, Pulmonary Embolism; AM, Adenomyosis; CIN III, Cervical Intraepithelial Neoplasia-III; TLH, Total Laparoscopic Hysterectomy; TAH, Total Abdominal Hysterectomy.

Additionally, the authors compared women with AM who underwent different surgical procedures. DVT incidence rates were as follows: laparoscopic lesion excision, 18.46 % (12/65); open lesion excision, 17.65 % (3/17); laparoscopic total hysterectomy, 7.86 % (25/318); and open total hysterectomy, 0.00 % (0/9). Regarding PE incidence rates, open lesion excision had the highest rate at 17.65 % (3/17), followed by laparoscopic lesion excision at 9.23 % (6/65), laparoscopic total hysterectomy at 1.89 % (6/318), and open total hysterectomy at 0 % (0/2). The incidence rates were also the highest in the open lesion excision group at 35.29 % (6/17), followed by laparoscopic lesion excision at 27.69 % (18/65), laparoscopic total hysterectomy at 9.75 % (31/318), and open total hysterectomy at 0 % (0/9) (Fig. 2, Fig. 3).

### Comparison of clinical characteristics between AM women with and without VTE

In this study, among the 411 women diagnosed with AM, 42 developed VTE (including two cases identified preoperatively), while 369 remained free of VTE. A comparative analysis of the clinical characteristics of the two groups revealed that the preoperative d-dimer levels were significantly elevated in the VTE group compared to the non-VTE group, demonstrating a statistically significant difference (*p* < 0.05). In contrast, no statistically significant differences were observed between the two groups concerning age, number of pregnancies, number of live births, number of miscarriages, uterine volume, BMI, Hb, PLT, PT, TT, APTT, or FIB (*p* > 0.05) (Table 3).

**Table 3:** Comparison of clinical data of patients with VTE and non-VTE.

Clinical features	VTE group	Non-VTE group	Statistic	p
Age (years, x̄ ± *s*)	46.40 ± 6.56	46.36 ± 5.10	*t* = −0.049	0.658
Pregnancy ([M (P25‒P75)])	3.0 (2.0‒4.0)	3.0 (2.0‒4.0)	*Z* = −0.601	0.548
Yield ([M (P25‒P75)])	1.0 (1.0‒2.0)	1.0 (1.0‒2.0)	*Z* = −1.050	0.294
Abortions ([M (P25‒P 75)])	1.5 (1.0‒2.0)	2.0 (1.0‒3.0)	*Z* = −0.141	0.888
Uterine Volume [cm^3^, (P25‒P75)]	524.0 (351.7‒724.6)	402.4 (261.7‒540.0)	*Z* = −1.708	0.088
BMI (kg/m^2^, x̄ ± *s*)	23.6 ± 2.1	23.8 ± 2.9	*t* = 0.719	0.848
Hb [g/L, M (P25‒P75)]	101.5 (85.0‒120.0)	107.0 (88.0‒123.0)	*Z* = −1.420	0.156
PLT [109/L, M (P25‒P75)]	261.5 (234.0‒323.5)	257.0 (211.5‒311.0)	*Z* = −1.166	0.244
TT [s, M (P25‒P75)]	14.9 (14.3‒15.8)	15.3 (14.5‒16.0)	*Z* = −0.802	0.423
PT [s, M (P25‒P75)]	11.5 (10.9‒12.0)	11.5 (11.0‒12.1)	*Z* = −1.246	0.213
APTT [s, M (P25‒P75)	28.9 (26.4‒31.1)	29.7 (27.8‒31.6)	*Z* = −1.822	0.068
FIB [g/L, M (P25‒P75)]	2.5 (2.3‒2.8)	2.6 (2.3‒2.9)	*Z* = −1.033	0.302
Preoperative D2 [µg/mL, M (P25‒P75)]	0.38 (0.23‒0.48)	0.27 (0.19‒0.41)	*Z* = −2.211	0.027

VTE, Venous Thromboembolism; Hb, Hemoglobin; PLT, Platelet Count; PT, Prothrombin time; APTT, Activated Partial Thromboplastin time; FIB, Fibrinogen; TT, Thrombin Time; BMI, Body Mass Index.

### Impact of different thromboprophylaxis measures on the incidence of VTE in women with AM post-surgery

Among the 409 women with AM who underwent surgical treatment, 328 received a combination of mechanical and pharmacological prophylaxis, resulting in a VTE incidence of 7.62 % (25/328). Conversely, the mechanical prophylaxis group, comprising 81 women, had a VTE incidence of 18.52 % (15/81). This difference was statistically significant (*p* < 0.05). Compared with the mechanical prophylaxis group, the combination of mechanical and pharmacological prophylaxis was associated with a significantly reduced incidence of VTE (7.62 % [25/328] vs. 18.52 % [15/81], *p* < 0.05) (Table 4).

**Table 4:** The effect of different preventive measures on the VTE patients with AM.

Group	VTE	Non-VTE	Total	Incidence
Mechanical + Drug prevention group	25	303	328	7.62 %
Mechanical prophylaxis group	15	66	81	18.52 %
Total	40	369	409	9.78 %

VTE, Venous Thromboembolism; AM, Adenomyosis.

## Discussion

AM predominantly affects women of reproductive age, with a reported incidence of 34 %.^28^ Typical clinical manifestations include the progressive worsening of secondary dysmenorrhea, menstrual irregularities, uterine enlargement, and infertility.^29^ The present study indicates that women with AM have lower age, number of pregnancies, number of live births, and Hb compared to non-AM women, while their uterine volume is significantly larger, consistent with existing literature. A study involving 43,751 women reported an overall incidence of VTE of 0.2 % following gynecological surgery, with a rate of 0.7 % specifically for hysterectomy.^30^ The incidence of VTE in women with malignant tumors is 12 times higher than that in women with non-malignant tumors, with DVT rates reaching as high as 11.4 %–30.8 %.^31^^,^^32^ In the present study, the incidence of postoperative VTE in the AM group was 13.45 %, which was significantly higher than the 6.06 % observed in the non-AM group and notably higher than the rates reported in the literature.

Several studies have suggested that women with AM may have intrinsic coagulation abnormalities attributable to increased uterine volume and recurrent abnormal uterine bleeding. Research indicates that AM leads to significant uterine enlargement, which may compress the iliac veins and slow blood flow. When combined with a postoperative hypercoagulable state, this can trigger thrombus formation.^33^ Yamanaka et al. found that when the uterine volume in women with AM exceeds 200 cm^3^, levels of thrombin-antithrombin complex increase, placing the blood in a hypercoagulable state, thus elevating the risk of thrombosis.^34^ Hong et al. demonstrated that as the uterine volume increases, inflammation and bleeding within AM lesions intensify, leading to increased consumption of coagulation factors in the extrinsic pathway, prolonged PT, and an elevated risk of thrombotic-related diseases.^22^ Among over 30 AM women with cerebral infarction, Disseminated Intravascular Coagulation (DIC), and VTE, 77.4 % had uterine enlargement. Furthermore, approximately 40 %–50 % of women with AM exhibit clinical symptoms of prolonged menstrual periods and increased menstrual flow, likely associated with increased endometrial surface area and enhanced vascularity of the endometrial basal layer.^35^^,^^36^ Abnormal uterine contractions, aberrant angiogenesis, elevated levels of various angiogenic factors, α-smooth muscle actin, endothelial glycoproteins, S100A13, profilin, Matrix Metalloproteinases (MMPs), Nuclear Factor (NF)-κB, Tissue Factor (TF), and transforming growth factor-β1 are among the factors contributing to the development of anemia in AM women.^37^ Anemia induces reactive increases in platelets and red blood cells, disrupting the expression of endothelial adhesion molecules, which places the body in a hypercoagulable state.^38^ In the present study, AM women exhibited higher PLT and postoperative d-dimer levels compared to non-AM women, likely due to compensatory increases in PLT production by the bone marrow to maintain normal physiological function in the context of anemia. The surgical approach may influence the incidence of VTE. Abnormalities in coagulation function do not necessarily lead to an increased incidence of postoperative VTE. The authors compared various clinical characteristics between patients who developed VTE and those who did not, and the results indicated that an increased uterine volume was not associated with the occurrence of postoperative TVE.

Some studies have indicated a higher incidence of DVT after open gynecological surgeries than after laparoscopic procedures.^39^^,^^40^ However, opposing views suggest that pneumoperitoneum associated with laparoscopic surgery may impede venous return from the lower extremities, thereby increasing the risk of VTE.^33^ The present findings indicate that the incidence of VTE in women with AM undergoing laparoscopic lesion excision was higher than that associated with other surgical methods, potentially because of the larger and denser nature of AM lesions, which may prolong the surgical duration and result in greater intraoperative bleeding and more challenging suturing.

The literature reports that the incidence of DVT can be reduced to between 0.4 % and 13.3 % using various prophylactic measures.^41^, ^42^, ^43^, ^44^, ^45^, ^46^ In this study, all women underwent thromboprophylaxis 12 h postoperatively. The incidence of VTE in the mechanical plus pharmacological prophylaxis group was 7.62 %, while the mechanical prophylaxis group had a VTE incidence of 18.52 %, which exceeds reported levels in the literature. In this study, the authors used only mechanical prophylaxis in women undergoing lesion excision because of the risk of active bleeding postoperatively. Although pharmacological prophylaxis can reduce the risk of VTE by approximately 50 %, it may simultaneously increase the risk of bleeding by approximately 50 %.^47^, ^48^, ^49^ For most women with AM who are already anemic, further bleeding poses a significant risk.

### Limitations

The limitations of this study include its single-center, retrospective design and relatively small sample size, necessitating further large-scale, multicenter, prospective epidemiological investigations to enhance the accuracy of the findings. The postoperative follow-up for VTE was limited to seven days, potentially overlooking some cases of delayed VTE. Additionally, incomplete collection of clinical data resulted in the exclusion of certain serological markers, such as Carbohydrate Antigen 125 (CA125), Carbohydrate Antigen 199 (CA199), Prothrombin Time-International Normalized Ratio (PT-INR), and Procalcitonin (PCT) from this study. Future studies should aim to comprehensively collect and analyze these markers to further explore the underlying causes of thrombotic events in postoperative women post-surgery. Finally, it is important to emphasize that this study does not provide sufficient evidence to recommend that all patients undergoing laparoscopic resection of adenomyosis lesions should undergo Doppler ultrasound examinations. In fact, further research are needed to validate the necessity of using Doppler ultrasound in this patient population.

## Conclusion

The present study indicates that women with AM experienced fewer pregnancies and births exhibited lower hemoglobin levels, and had larger uterine volumes compared to women without AM. Additionally, the preoperative platelet counts and postoperative d-dimer levels were significantly elevated in women with AM. The incidence of VTE after surgery was markedly higher in women with AM than in those without, with a greater incidence of VTE following laparoscopic lesion excision compared to laparoscopic hysterectomy. Furthermore, the authors found that a combination of mechanical and pharmacological prophylaxis may be more effective in preventing VTE than mechanical prophylaxis alone. Finally, considering that women with AM may experience asymptomatic VTE postoperatively, it is advisable for all patients to undergo duplex ultrasonography of the lower extremities within 7 days after surgery, regardless of the presence of VTE symptoms.

## Data sharing statement

The data that support the findings of this study are available on request from the corresponding author Yan Lei upon reasonable request.

## Ethics statement

This study involves human participants. Ethical clearance and support letters were obtained from the Ethical Committee of Maternal and Child Health Hospital of Hubei Province with the reference number IEC042. The methods were conducted following the tenets of the Helsinki Declaration. Before collecting data, an information sheet was read to all eligible study participants to obtain oral informed consent. The privacy of the respondents was respected, and data were deidentified before analysis and reported in aggregate. Participants gave informed consent to participate in the study before taking part.

## Funding

No project or corporate funding was available to support this study.

## CRediT authorship contribution statement

**Yan Lei:** Conceptualization, Methodology, Software, Writing – original draft, Writing – review & editing. **Na Chen:** Data curation, Validation. **Yuqin Tang:** Visualization, Investigation. **Xiaojia Xie:** Supervision, Software.

## Declaration of competing interest

The authors declare no conflicts of interest.

## Appendix Supplementary materials

Image, application 1

Image, application 2

Image, application 3

Image, application 4
