# Supplementary material for: Different prophylactic measures for preventing postoperative deep venous thromboembolism in adenomyosis: a retrospective study
Source: Clinics (Sao Paulo). 2025 Jun 14;80:100700. doi: 10.1016/j.clinsp.2025.100700 (PMC12206018; doi:10.1016/j.clinsp.2025.100700)
Supplement: Supplementary file 1 [file mmc1.docx]

**CRediT author statement**

Yan Lei: Conceptualization, Methodology, Software Writing- Original draft preparation,Writing- Reviewing and Editing.

Na Chen: Data curation , Validation.

Yuqin Tang: Visualization, Investigation.

Xiaojia Xie: Supervision,Software.
