# Supplementary material for: Different prophylactic measures for preventing postoperative deep venous thromboembolism in adenomyosis: a retrospective study
Source: Clinics (Sao Paulo). 2025 Jun 14;80:100700. doi: 10.1016/j.clinsp.2025.100700 (PMC12206018; doi:10.1016/j.clinsp.2025.100700)
Supplement: Supplementary file 3 [file mmc3.docx]

Yan Lei: Conceptualization, Methodology, Software, Investigation, Formal Analysis, Writing - Original Draft,Writing - Review & Editing

Na Chen: Data Curation, Visualization

Yuqin Tang: Resources, Supervision

Xiaojia Xie: Software, Validation ,Conceptualization
