# Supplementary material for: Different prophylactic measures for preventing postoperative deep venous thromboembolism in adenomyosis: a retrospective study
Source: Clinics (Sao Paulo). 2025 Jun 14;80:100700. doi: 10.1016/j.clinsp.2025.100700 (PMC12206018; doi:10.1016/j.clinsp.2025.100700)
Supplement: Supplementary file 2 [file mmc2.docx]

Dear Editor,

Thank you for your review and feedback on our manuscript. Regarding your comment about the number of references, we understand that an excessive number of references can sometimes affect the clarity and readability of the article.

We have 51 references in our manuscript primarily to ensure the comprehensiveness and accuracy of the topic discussed. Here are the reasons we believe it is important to retain these references:

1.Breadth of Literature: Our research spans multiple relevant fields, and we have cited literature from various disciplines to provide a more comprehensive background and support for our findings.

2.Diversity of Supporting Research: The references encompass different methodologies and results, which help readers understand the foundation and significance of our study.

3.Inclusion of Recent Advances: We have ensured that the references include the latest research findings to reflect the most current developments in the field.

If you still feel that a reduction in the number of references is necessary, we will make adjustments according to your suggestions. Thank you for your attention and support for our work.

Sincerely,

Yours,

Yan Lei
